# A proposed redesign of elective cataract services in Scotland – pilot project

**DOI:** 10.1038/s41433-021-01810-9

**Published:** 2021-10-22

**Authors:** Niku Dhillon, Dina Ghazal, Jane Harcourt, Manjula Kumarasamy

**Affiliations:** Department of Ophthalmology, Aberdeen Royal Infirmary, Scotland, AB25 2ZN UK

**Keywords:** Lens diseases, Public health

## Abstract

**Background:**

The demand for cataract surgery is expected to increase by 25% in the next 10 years as the result of our ageing population. A new pathway is being proposed to improve efficiency by utilising the new General Ophthalmic Services code 2.9 for community optometrists in Scotland for Cataract Referral Refinement and Consenting process. A pilot project has been undertaken at NHS Grampian enabling patients to be assessed and undergo surgery at a single visit to the Eye Outpatient Department.

**Objectives:**

To determine the suitability of community cataract referrals for a one stop cataract surgery service and target areas for referral refinement.

**Methods:**

300 consecutive cataract referrals were assessed for suitability for one stop cataract surgery, determined by the documentation of pertinent clinical findings. All suitable referrals were offered a telephone consultation to confirm suitability and those patients were subsequently offered on the day cataract surgery. A telephone led patient satisfaction survey was then completed.

**Results:**

71 (24%) suitable patients were identified from vetting 300 referrals. 54 patients from this group were selected for one-stop service following telephone consultation. 51 patients subsequently attended for surgery. There was a 100% conversion rate to same day surgery and no intraoperative complications reported.

**Conclusion:**

The waiting time was significantly reduced, by 30 weeks, for one-stop patients. Approximately one quarter of referrals were deemed suitable for a one-stop service. Many more patients may have been suitable for same day surgery but there was not sufficient information in their referrals to determine their suitability.

## Introduction

With an ageing population and increasing pressures on an already overburdened health service innovative measures are needed to optimise existing resources. The cataract referral pathway in the UK has been streamlined over the past two decades from a 5-step to a 2-step process. Nonetheless, as the demand for cataract surgery continues to grow a more focused model is required.

The Action on Cataracts [1] guidance reported a 2–3 month wait for an initial pre-assessment appointment followed by a further seven months for surgery. Cataract surgery is the most commonly performed operation in the UK with over 400,000 performed in England and Wales between 2017 and 2018 [2] and the demand is predicted to increase by 50% over the next twenty years [3]. With finite resources the existing cataract pathway needs to be adapted to reduce waiting times and pressure on both community and hospital eye services (HES).

Previously, the five-step process would involve an appointment with an optometrist, a general practitioner to facilitate the referral, a pre-assessment with an ophthalmologist, a biometry appointment and a date for surgery. Postoperative care of an uncomplicated procedure would require a further postoperative check and updated refraction. All steps require specialist resources in an overstretched health service, transport costs, patient anxiety, time off work and family-related disruption. Any savings would be of great economic and financial benefit.

A one stop pathway involves a process where low risk patients are selected from a detailed community referral and offered a single half day hospital appointment for biometry, assessment, consent and cataract surgery. One stop surgery is routine practice at the University Hospital of Ayr, Scotland since 2001 and demonstrates that a one stop model is a feasible cost-effective alternative [4]. Ayrshire’s model is dependent on the referral from a specialist optometrist with additional accreditation in the assessment of cataract.

**Fig. 1:**
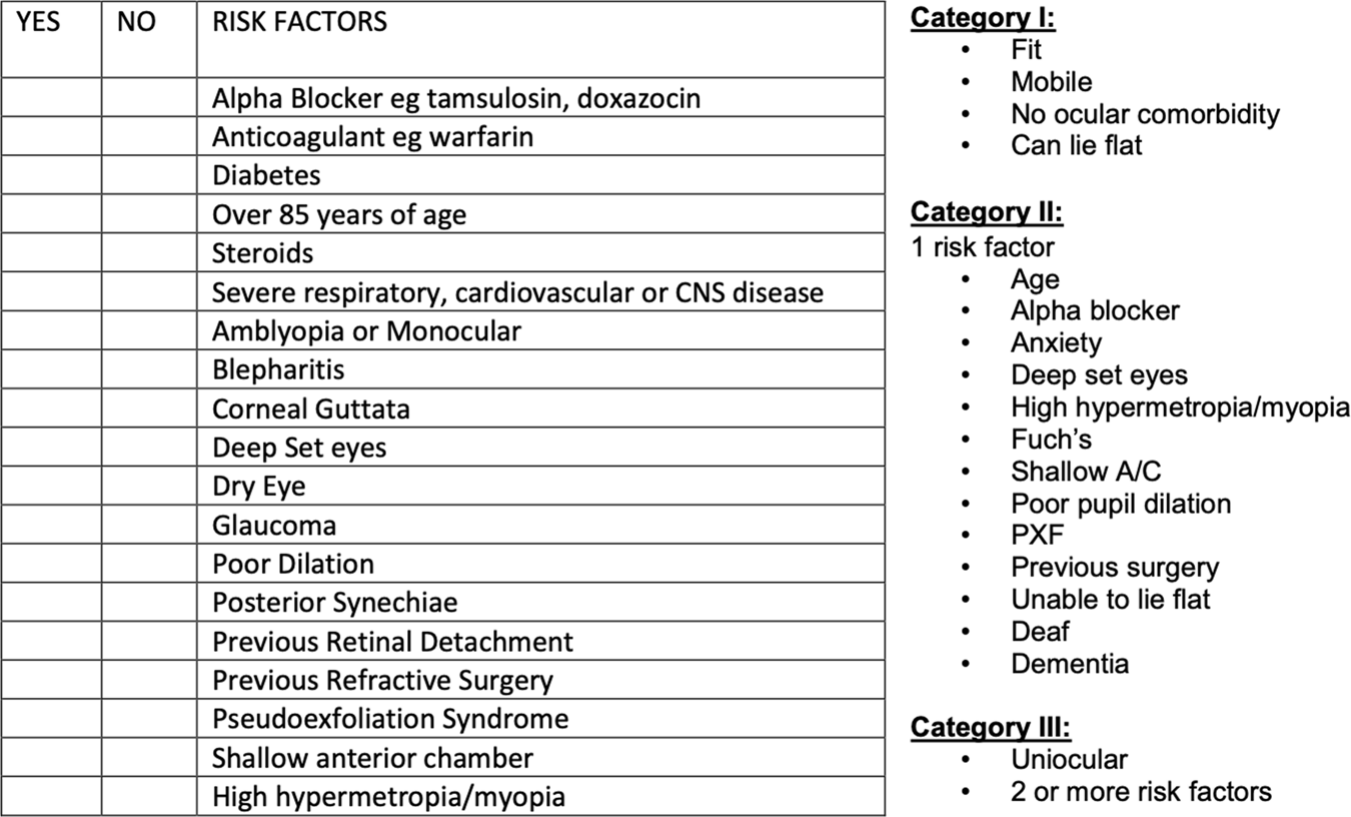
Code 2.9 Aide Memoire: ‘Cataract referral advice and counselling’ and risk stratification.

We propose a one stop service could be delivered by general optometrists using the 2018 Scottish GOS supplement code 2.9 for the provision of cataract referral and counselling. The accompanying aide memoire (Fig. 1) aims to identify the appropriate patient, optimise referral quality and initiate the consent process.

The study aims are four-fold

- To determine the suitability of community cataract referrals for a one stop cataract surgery service
- Target areas for referral refinement
- Determine the safety and efficacy of a one stop cataract surgery service.
- To determine patient satisfaction following experience of the one stop cataract service model

## Methods

In a pilot study 300 consecutive community cataract referrals to NHS Grampian hospital eye service at Aberdeen Royal Infirmary, between September 2018 and October 2018 were assessed by a senior ophthalmologist for suitability for pre-assessment, biometry and cataract surgery on the same day, “one stop cataract surgery”. A sample size of 300 referrals was chosen as this number constituted one third of the total cataract waiting list.

All referrals were assessed to determine whether the following information had been included: symptoms, past ocular history, past medical history, drug history, appropriate level of vision (equal to or worse than 6/9.5 or 0.2 logMAR), refraction details, intraocular pressure (IOP), health of eyelids and cornea, pupil size after dilation, presence of pseudoexfoliation, density of cataract and retinal appearance.

The documentation of relevant positive and negative findings, outlined by Fig. 1, are important in a community referral in order to appropriately risk stratify patients for cataract surgery. The Code 2.9 aide memoir, used by community optometrists under the GOS contract, is adapted from Gupta et al.’s cataract classification system [5]. Patients identified as category one or low risk for posterior capsular rupture were offered same day cataract surgery and issued with information documents (Appendix 1). These included an appointment for a telephone consultation plus a cataract surgery information leaflet along with the consent form for cataract surgery, preoperative eyelid hygiene, preparing for eye surgery, eye surgery aftercare and how to use eye drops.

The telephone consultation gave the opportunity to further discuss the surgery with a consultant ophthalmologist, outline preoperative and postoperative care, identify any overlooked factors that may increase the risk for more complicated surgery and verbally consent the patient for same day surgery. Approximately 20 min of clinical time was required to complete the consultation and documentation.

All patients enroled in the pilot project were given a date for surgery and operated on by a second consultant ophthalmic surgeon at a single visit to the Eye Out Patient Department at Aberdeen Royal Infirmary. Three study patients and three standard pathway patients were booked onto each standard non-training service provision cataract list over a period of 6 weeks. Patients were staggered throughout the session and two hours prior to their surgery to allow time for an ophthalmic nurse to take visual acuities, perform biometry, instil dilating drops and complete the necessary paperwork required for cataract surgery. The surgeon then completed their slit lamp assessment, consent procedure, selected a lens and marked the eye.

Intraoperative and postoperative complications were recorded. Postoperative visual acuities were obtained from refraction feedback forms returned by the community optometrists following the patient’s 6-week postoperative assessment.

Following the surgery all patients received a telephone led patient satisfaction survey.

## Results

Of 300 consecutive cataract referrals 295 (98%) were referred by the patient’s community optometrist and 5 (2%) by their GP. 71 (24%) referrals were considered low risk and therefore suitable for the one stop cataract surgery model. Of the suitable referrals (*n* = 71) 63% were female (*n* = 45) and the median age was 74 years (range 48–94). Table 1 compares the unsuitable and suitable cataract referrals for the mention of relevant information in order to risk stratify cataract surgery.

**Table 1:** Comparison between unsuitable and suitable community cataract referrals.

Criteria	Unsuitable referrals for same day surgery (*n* = 229)	Suitable referrals for same day surgery (*n* = 71)	Difference between groups (*p* value)
Refraction	225 (98%)	70 (99%)	0.85 (χ² = 0.04)
Symptoms	188 (82%)	64 (90%)	0.11 (χ² = 2.61)
Past ocular history	116 (50%)	60 (84%)	4.17 × 10^−7^ (χ² = 25.61)
Drug history	80 (35%)	66 (92%)	1.27 × 10^−17^
Past medical history	113 (49%)	67 (94%)	1.33 × 10^−11^ (χ² = 45.77)
Appropriate vision (≥6/9.5)	205 (90%)	62 (87%)	0.61 (χ² = 0.27)
Intraocular pressure	189 (83%)	60 (85%)	0.70 (χ² = 0.15)
Eyelids	80 (35%)	46 (64%)	8.47 × 10^−6^ (χ² = 19.83)
Cornea	64 (28%)	34 (47%)	0.002 (χ² = 9.80)
Pupil size after dilation	1 (0.4%)	0 (0%)	0.58 (χ² = 0.31)
Pseudoexfoliation	0 (0%)	0 (0%)	1 (χ² = 0)
Density of cataract	191 (83%)	69 (97%)	0.003 (χ² = 8.90)
Appearance of retina	181 (79%)	69 (97%)	3.3 × 10^−4^ (χ² = 12.85)

**Fig. 2:**
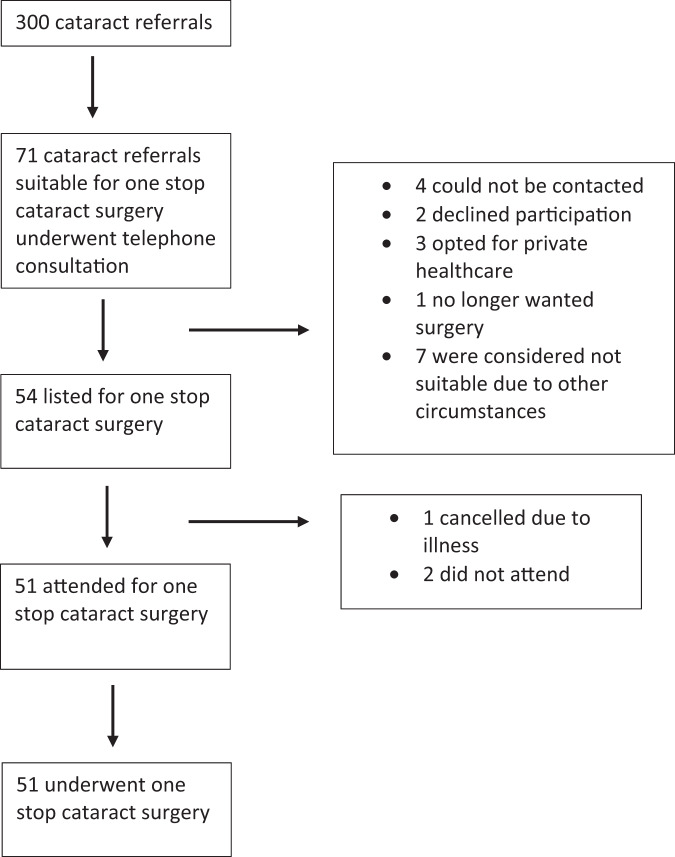
Selection process for suitable patients to undergo one stop cataract surgery.

After contacting suitable patients, 54 patients were listed for one stop surgery (see Fig. 2). One patient could not attend their date of surgery due to illness and two did not attend.

The median wait time from referral to surgery was 21.4 weeks (range 18.9–37.4). Of 51 individuals who attended for surgery 51 (100%) cataract surgeries were performed. 57% were female (*n* = 29) and the median age was 74 years (range 49–89 years). Of the operated eyes the preoperative median visual acuity was 0.30 logMAR (range 0.00–1.00).

There were no posterior capsular ruptures or other intraoperative complications. There was one persistent uveitis at the 6-week postoperative review and two cases of cystoid macular oedema. One resolved spontaneously and one required treatment with a topical non-steroidal anti-inflammatory.

**Fig. 3:**
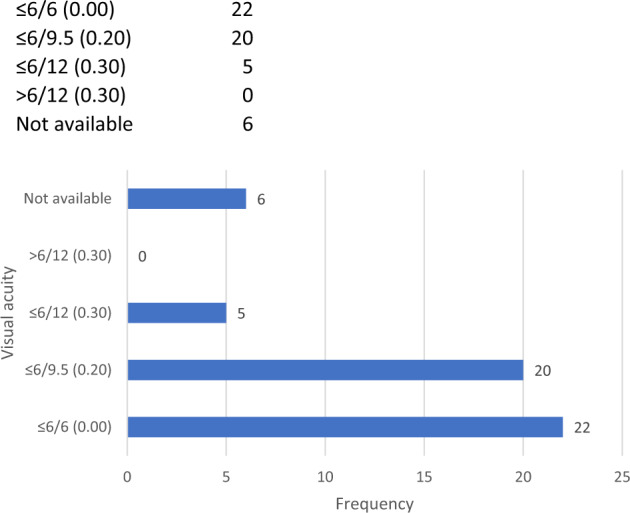
Postoperative corrected visual acuity outcomes.

47 (89%) postoperative refractions were completed and returned. The median corrected distance visual acuity was 0.10 logMAR (range −0.10–0.30) (see Fig. 3).

46 (90%) of 51 patients were successfully contacted to complete a telephone questionnaire. 4 could not be contacted and 1 patient had died. Overall satisfaction was very high and all patients would recommend the process to a friend Table 2.

**Table 2:** Results of the telephone led patient satisfaction questionnaire.

Question	Strongly disagree (1) to Strongly agree (5) Mean value, *n* = 46
Did the cataract information letter provide adequate information to prepare you for surgery?	4.8
Was the cataract information letter clear?	4.8
Were you satisfied with the telephone consultation?	4.9
Were you prepared/happy turning up on the day of your surgery?	4.8
On the day of surgery did you feel it was a smooth process?	4.7
On the day of surgery did you feel rushed?	4.7
On the day of surgery were you satisfied with how your doctor consented you for surgery?	4.8
Were you made comfortable during your surgery?	4.8
Were the discharge instructions clear when you were discharged after your surgery	4.9
Overall experience	4.9
Would you recommend the 1 stop process to a friend considering cataract surgery?	4.9

## Discussion

This pilot project aimed to determine the feasibility of a one stop cataract service in NHS Grampian. 24% of community referrals were considered suitable for one stop cataract surgery. Many referrals lacked general information including past ocular, past medical and drug history, all of which information is required for risk stratification of surgery. A detailed examination of the external eye and fundus is a fundamental requirement before considering cataract surgery, and these details are frequently not documented. Pupil size after dilatation and the presence of pseudoexfoliation are not routinely documented in the community; however, these findings are essential when planning surgery. Lash et al. suggest that less than one fifth of general ophthalmic service (GOS) referrals have the required information [6].

As of October 2018, a supplementary eye examination in primary care is funded by the Scottish GOS contract [7]. Code 2.9 offers optometrists an additional £24.50 for the provision of cataract referral advice and counselling following an initial eye exam. This may include providing prognosis or counselling and preparation for consent for cataract surgery, including risk factors [7]. General optometrists have the skills to identify and document pertinent ophthalmic findings without additional specialist cataract accreditation. Although, further training and awareness of what information is needed within the electronic referral with mandatory fields may improve the referral quality and facilitate one stop surgery.

As this was a new initiative within the health board an experienced cataract surgeon was involved in the rigorous vetting of referrals and telephone pre-assessment in order to achieve accurate and appropriate listing of patients for cataract surgery. The telephone consultation was necessary to obtain a more comprehensive description of the patient’s functional status in order to accurately risk stratify. Details including reduced mobility and inability to lie flat are often omitted from the community referral and can be clarified without the patient coming to hospital. Now that the pilot project has been shown to be a safe and effective alternative pathway within the health board these consultations can be delivered by other appropriately trained eye care professionals.

Fully informing the patient during the telephone consultation was key to both achieving a 100% conversion to same day surgery and ensuring the surgeon’s expended time during preoperative assessment was not much different to any other routine case. There were no intraoperative complications and the rate of postoperative complications appears comparable to standard cataract surgery [2]. Postoperative visual acuities were within national recommended outcomes [2]. The patient satisfaction for the one stop model was reported as very high. One patient was dissatisfied with the process on the day of surgery, however rated the overall experience highly.

Good communication is fundamental for the co-management of cataract patients between the community and hospital eye service. Optometrists have the skills to undertake effective cataract assessments and such a resource should be utilised to streamline the cataract pathway. It is commonplace to discharge uncomplicated postoperative cataracts on the day of surgery to a community optometrist. Renumeration for this service agreement is provided on return of the results of the 6-week postoperative refraction as per code 2.7 of the GOS contract. With regard to the assessment of postoperative complications Revicki et al. demonstrated that optometrist diagnosis of postoperative complications was accurate [8] enabling effective treatment and avoidance of unnecessary hospital visits.

To ensure appropriate use of time and resources schemes have been set up for additional optometrist training to perform cataract pre-assessments. Bowes et al. created an annual accreditation course for Cambridgeshire optometrists in an effort to improve the quality of referrals to ophthalmology [9]. And Gaskell et al. [4] developed a specialist educational course to accredit optometrists in the assessment of cataract. Our data suggest that referrals from community optometrists should include information regarding co-existing ocular and medical pathology. An Aide Memoir accompanies the code 2.9 supplement for cataract risk stratification and authorises a general optometrist to initiate the one stop cataract pathway for the correct patient (Fig. 1).

In order to comply with social distancing measures in the post-COVID era, eye health professionals should strive to ensure that patients are not referred unnecessarily as once the pathway is initiated it may cause undue patient anxiety and place vulnerable people at risk of avoidable exposure. The Action on Cataracts project [1] and The National Eye Care Services Steering Group [10] introduced three questions which aim to correctly initiate the cataract pathway for the correct patient:

- Is cataract the main cause of reduced visual acuity?
- Is there a significant impact on lifestyle?
- Is the patient agreeable to proceed with surgery?

These prompts encourage discussion and involve the patient early in the shared decision-making process. It is often clear if a patient does not wish to undergo cataract surgery thereby avoiding a hospital appointment and reducing pressure on hospital resources.

NICE guidelines recommend primary care health providers to counsel patients on cataract surgery when the referral is made [11]. 97% of optometrists compared to 18% of GPs had a discussion regarding the risks and benefits of cataract surgery [12]. This was reflected in the listing rates with 87% of optometrist referrals proceeding to surgery compared to 69% of GP referrals.

The inclusion of a focused medical and ophthalmic history and examination is fundamental to the cataract pre-assessment. Past medical history, a frequently overlooked item in optometrist referrals is important in the identification and optimisation of modifiable risk factors. Park et al. observed 68% optometry referrals included past medical history compared to 94% of GP referrals [12]. Documentation of a drug history with particular regard to blood thinners and alpha blockers are important during surgical planning.

Pre-assessment allows the opportunity to screen for and identify systemic health conditions. Cataract operations performed under local anaesthetic do not undergo general health pre-assessment and medical co-morbidity can be overlooked. Prasad et al. demonstrated that a large proportion of their cancelled cataracts were as a result of uncontrolled hypertension [13]. Knowledge of a patient’s past medical history could be used to target at risk populations and optimise their general health prior to surgery.

Prasad et al. document that the pre-assessment visit is considered to be a valued opportunity [13] for the patient to discuss the procedure and for the listing doctor to facilitate a patient centred approach. Nonetheless, in the current climate a face-to-face appointment may no longer be required, where an information leaflet or telephone call may suffice.

Shankar’s comment [14] highlighted the importance of a physical appointment to discuss surgery with medical staff. Although patients were supplied with an information letter, a small proportion declined surgery on the day after discussion with an ophthalmologist [15]. It is possible patients may feel pressured into same day surgery and may benefit from the opportunity to discuss and in some cases seek reassurance from an ophthalmologist before committing to surgery. This opportunity is provided during the telephone consultation.

A low conversion rate of appointments to completed operations is a primary concern with regard to a one stop model resulting in empty theatre time and poor utilisation of skilled healthcare staff. Hughes et al. mentioned that unexpected ocular findings and cancellations are inevitable [15]. Ocular co-morbidity, insufficient cataract and vision not significantly impacting on daily activities were reasons for not listing for cataract surgery [6]. Our study demonstrated that through careful patient selection a conversion rate of 100% was achieved. The surgical team remained consistent throughout the project facilitating efficiency. This surgeon’s threshold for operating may have contributed to the high rate of conversion.

Evans et al. [16], Hughes et al. [15] and Gaskell et al. [4] estimated a listing rate of 56.6%, 82.1% and 96%, respectively, using their one stop day surgery model. The difference being Gaskell et al. delivered a specialist educational course for referring optometrists to ensure referrals were of high quality and suitable patients for the pathway received a cataract specialist nurse led telephone consultation. We propose that code 2.9 of the Scottish GOS contract and accompanying Aide Memoir (Fig. 1) will permit non-specialist optometrists to identify low risk patients and initiate the consent process within the primary care setting.

The GMC states that the decision to undergo any procedure is an ongoing process. In our pathway we propose that the consent process for cataract surgery starts at the point of contact with the community optometrist under code 2.9. Prior to referral onto HES the patient will be issued both information on cataract surgery and the consent document. The patient will be offered a subsequent opportunity to discuss the procedure with their community optometrist and once agreeable the referral onto HES would be initiated. There will be two further points of contact with the hospital eye services: the telephone consultation and on the day of surgery. At these temporally separated points in the pathway, the dialogue continues allowing for ample reflection time, confirmation of understanding and a further opportunity to consider the patient’s wishes, for example, target refraction.

Although the one stop model is completed in a more time efficient manner, it was highlighted that the absolute workload and resources required are similar [15]. It is difficult to establish whether patient satisfaction is higher in a one stop service compared to standard practice as there is no national standard and secondly to accurately assess patient satisfaction between models, patients would be required to undergo consecutive cataract surgery using both the standard and one stop model for each eye. In keeping with our results, Hughes et al. [15] and Gaskell et al. [4] have suggested that patients were satisfied with their experience of the fast track cataract pathway.

Limitations of the study include the retrospective data collection and single-centre design. The patient satisfaction responses were conducted via telephone interview one year after the operation, opening the risk to recall bias. Final visions and presence of postoperative complications were determined from the 6-week refraction. A longer follow-up period could be completed to determine the long-term safety of the one stop model. Although the NICE guidelines [11] for cataract surgery suggest visual acuity should not restrict access to surgery, the cut off in this study for appropriateness of referral was a vision of equal to or worse than 6/9.5 or 0.20 logMAR.

To apply the model, a pre-assessment educational course for community optometrists would be advantageous. Using the Aide Memoire general community optometrists should be able to determine which patients are suitable to follow the one stop pathway and subsequently initiate the process of consent. Only low risk patients would be considered suitable for the one stop service. More complex patients would follow the conventional hospital pre-assessment route. An administrative co-ordinator would be required to book appointments and send out the pertinent information documents.

Once the pathway is established in practise, the delivery of the telephone pre-assessment can be transferred to an allied eye health professional with the involvement of a hospital optometrist or specialist cataract nurse. On the day of surgery, a designated ophthalmic nurse would perform the biometry, instil dilating drops and complete the necessary paperwork required for cataract surgery. The operating surgeon would examine the patient, select the lens power and complete the consent process. Following surgery, a nurse from the surgical team would discharge the patient with postoperative drops, the 6-week refraction feedback form and signpost the patient to contact the specialist cataract nurse phone number if any problems arose.

## Conclusion

We suggest that one stop cataract surgery would be suitable for low risk patients particularly those who are required to travel long distances. It complies with COVID risk mitigation measures by halving the number of appointments and reduces the wait for cataract surgery. Excellent patient satisfaction survey results would suggest that the pathway is acceptable to patients.

Looking ahead, the feasibility of a one stop model would be dependent on the development of an educational online tutorial for general optometrists on how to conduct a focused cataract history, examination and discussion in order to identify an appropriate candidate for one stop surgery and optimise referral quality. With appropriate training the initial community assessment could replace the hospital-based cataract pre-assessment thereby reducing waiting times at no additional cost to the health board. Using careful patient selection and an experienced ophthalmic surgeon a one stop community to cataract surgery model appears to be a safe and effective use of resources.

### Summary

#### What was known before

- The demand for cataract surgery is expected to rise and resources are becoming increasingly limited.
- The number of hospital visits should be minimised in order to comply with social distancing measures.

#### What this study adds

- A proposed fast track one stop referral pathway for uncomplicated cataract surgery can be initiated by general community optometrists using the 2018 Scottish General Ophthalmic Services (GOS) contract at no additional cost to the health board.
- A cataract pathway that complies with COVID risk mitigation measures.
- Approximately one quarter of cataract referrals had sufficient information to safely list for one stop cataract surgery. Many more uncomplicated cataract referrals may have been suitable if sufficient clinical information was documented.

## Supplementary information

Appendix 1
