# Supplementary material for: A proposed redesign of elective cataract services in Scotland – pilot project
Source: Eye (Lond). 2021 Oct 22;36(11):2116–21. doi: 10.1038/s41433-021-01810-9 (PMC9581890; doi:10.1038/s41433-021-01810-9)
Supplement: Supplementary file 1 — Appendix 1 [file 41433_2021_1810_MOESM1_ESM.pdf]

PATIENT INFORMATION LEAFLET

NHS Grampian

Integrated Cataract Assessment & Surgical Extraction Project.

You have been selected to take part in the pilot scheme for a direct cataract referral to same day surgery in the Eye Out-patient Department. This project is part of the on-going initiative to reduce the waiting time for patients undergoing cataract surgery in Grampian.

What is the purpose of this scheme?

The purpose of the project is **to combine the clinic appointment for cataract assessment and have operation on the same day**. Currently, patients referred for cataract surgery by optometrists are first seen in cataract clinic for a pre-operative assessment then wait for surgery to be scheduled on a later date. A new One-stop cataract service is being piloted where patients will be able to access same day diagnosis, assessment and operation at the new Eye Out-Patient Cataract Theatre facility in ARI. It will result in considerable reduction in the waiting time for cataract surgery.

What happens before your visit?

Enclosed with your appointment letter are the following documents:

- Consent for cataract surgery
- Preparation for eye surgery booklet
- How to use eye drops
- Eye surgery aftercare

A member of staff will phone you to discuss further before the appointment date. Please inform if you’re a contact lens wearer as you may not be suitable for same day surgery.

Please bring the following items with you to your appointment. Failure to bring any of the following may result in an incomplete or an inaccurate assessment.

• An up to date opticians prescriptions for any glasses that you are currently wearing (these can be obtained on request) as well as your glasses.
• A list of all the tablets, ointments, inhalers, insulin or eye drops that you are currently using. Please bring all medications.
• A urine sample.

• We advise that you arrange transport home; **You must not drive yourself home.**

• Eye Out Patients has a small area for patients to wait, due to lack of seating we may ask that relatives/friends not stay with you unless you require assistance with communication or mobility.

What will happen during my visit?

This appointment will last approximately 3 – 5 hours. You will attend ward 203 first to see a nurse and undergo tests and assessments. You will then be taken to the eye clinic operating theatre to see a consultant eye surgeon and make the decision to proceed with surgery. The operation details are informed in the Cataract information leaflet. You will be explained about after care by the nursing team.

What happens if I am not suitable for surgery on the same day?

Based on the information provided in the referral letter, we expect you to be suitable to undergo operation on the same day. However if the surgeon feels you're not fit for surgery for various reasons, for example, if your blood pressure is uncontrolled, have a sticky eye, further testing required etc then your operation will be postponed to another date.

Contact for further information

If you have any queries or not keen/unable to accept same day surgery please contact the project coordinator Pat Peacock on 01224 550201 or patricia.peacock@nhs.net

Lead Clinicians for the Integrated Cataract Assessment & Surgical Extraction Project are Miss Manjula Kumarasamy and Miss Jane Harcourt.

CONSENT FOR CATARACT SURGERY

INFORMATION FOR PATIENTS

This leaflet gives you information that will help you decide whether to have cataract surgery. You might want to discuss it with a relative or carer. Before you have the operation you will be asked to sign a consent form therefore it is important that you understand the leaflet before surgery.

THE CATARACT

You have been referred for cataract assessment or surgery because the lens in your eye has become cloudy, making it difficult for you to see well enough to carry out your usual daily activities.

If the cataract is not removed your vision may stay the same,but it may get worse. Waiting for a longer period of time could make the operation more difficult. Your eyesight may become so poor that all you can see is light and dark.

THE OPERATION

The purpose of the operation is to replace the cloudy lens(cataract) with a new clear plastic lens(implant)inside your eye. An experienced eye specialist will carry out the operation or may supervise a doctor in training who also perform some operations. With a local anaesthetic you will be awake during the operation. You will not be able to see what is happening, but you will be aware of a bright light. Just before the operation you will be given eye drops to enlarge the pupil. These may sting, this is normal. After this you will be given an anaesthetic to numb the eye. This may consist simply of eye drops or injecting a local anaesthetic solution into the tissue surrounding the eye.

During the operation you will be asked to keep your head still and lie flat as possible. The operation normally takes 15-20 minutes but may take up to 45 mins. A nurse will hold your hand throughout the procedure.

Most cataracts are removed by a technique called phacoemulsification, The surgeon makes a very small cut in the eye, softens the lens with sound waves and

removes it through a small tube. The back capsule of the lens is left behind. An artificial lens(implant) is then inserted into the remaining capsule. Sometimes a small stitch is required,at the end of the operation a pad or shield may be put over the eye to protect it.

AFTER THE OPERATION

If you have discomfort we suggest that you take a pain reliever such as paracetomol every 4-6 hours(NOT ASPIRIN as this can cause bleeding). It is normal to feel itchy, sticky eyelids and mild discomfort for a while after cataract surgery. Some fluid discharge is common after 1-2 days even mild discomfort should disappear. In most cases healing will take about 2-6 weeks, after which new glasses can be prescribed by your optician. You will be given eye drops to reduce inflammation and prevent infection. The hospital staff will explain how and when to use them. Please do not rub your eye.

Certain symptoms could indicate that you need prompt treatment. Please contact the hospital immediately if you have any of the following symtoms.

:EXCESSIVE PAIN

:LOSS OF VISION

:INCREASING REDNESS OF THE EYE

LIKELIHOOD OF BETTER VISION

After the operation you may read or watch tv straight away but your vision may be blurred. The eye needs time to adjust so that it can focus properly with the other eye, especially if the other eye has a cataract. The vast majority of patients have improved eyesight following cataract surgery.

Please note that if you have another condition such as diabetes, glaucoma, age related macular degeneration your quality of vision may still be limited even after successful surgery.

BENEFITS AND RISKS OF CATARACT SURGERY

The obvious benefits are greater clarity of vision and improved colour vision. The lens implants are selected to compensate for existing focusing problems,most people find that their eyesight improves considerably after surgery,but will need to replace their glasses or single lens.

However you should be aware that there is a small risk of complications either during or after surgery.

SOME POSSIBLE COMPLICATIONS DURING OPERATION

Tearing of the back part of the lens capsule with disturbance of the gel inside the eye that may result in reduced vision.

Loss of all or part of the cataract into the back of the eye requiring a further operation which may need general anaesthetic.

Bleeding inside the eye.

SOME POSSIBLE COMPLICATIONS AFTER THE SURGERY

Bruising of the eye or eyelids

High pressure inside the eye

Clouding of the cornea

Incorrect strength or dislocation of the implant

Swelling of the retina-macular oedema

Detached retina which can lead to loss of sight

Infection in the eye-endopthalmitis which can lead to loss of sight or even the eye.

Allergy to the medication used

Complications are rare and in most cases treated effectively. In a small proportion of cases further surgery may be needed. Very rarely some complications can result in blindness.

The most common complication is called posterior capsular opacification. It may come on gradually after months or years. When this happens the back part of the lens capsule which was left in the eye to support the implant becomes cloudy. This prevents light from reaching the retina. To treat this the eye specialist uses laser beam to make a small opening in the cloudy membrane in order to improve the eyesight. This is a painless out patient procedure which normally takes only a few minutes.

We hope this information is sufficient to help you decide whether to go ahead with the surgery. Dont worry about asking questions as our staff will be happy to answer them.

Private & Confidential

NHS
Grampian

Patient Consent Form
for Clinical Procedures & Healthcare Interventions

Ophthalmology Cataract Extraction (Version 1.0)

Surname ▶

First Names ▶

Address ▶

Bar Code ▶

◀ CHI / Unit No

◀ Gender

◀ Date of Birth

◀ Post Code

PATIENT LABEL SHOULD BE USED IF AT ALL POSSIBLE
AND PLACED CAREFULLY WITHIN BRACKETS

Hospital/Location	
Responsible Clinician	
Ward / Dept	Age

- This form is to be used in association with the NHS Grampian 'Policy for Obtaining Consent for Clinical Procedures and Healthcare Interventions'
- Please review sections 1-6 and complete all that are applicable.

PLEASE FILE IN **SECTION B**

1

1a. Proposed procedure or treatment
(include brief explanation if medical term is not clear to patient/guardian/relative)

Cataract extraction with implant under local anaesthetic.

1b. Possible additional procedures which may be required
(include brief explanation if medical term is not clear to patient/guardian/relative)

Anterior vitrectomy.

2

Statement of health professional
I have explained the purpose of the procedure/treatment to the patient/child and parent/guardian/relative (delete as appropriate). In particular, I have explained:

2a. The intended benefits:

Improve vision.

2b. The known side effects and significant risks:

1 in 1000 risk blindness ☐

1 in 100 risk of operative complication requiring further surgery ☐

2c. The patient has been supplied with the following information (e.g verbal instructions, leaflets etc.)

Discussion ☐

Information leaflets ☐

2d. That during the course of the proposed procedure(s)/treatment, circumstance(s) may require an additional procedure(s) - see Section 1b above.

2e. I have also discussed alternative procedure(s)/treatments (including no treatment) and any particular concerns the patient may have.

I confirm this patient/parent/guardian has the capacity to give consent: YES ☐ NO ☐
(refer to Section 14 of policy)

Signed | Date

Name (PRINT) | Professional Reg. No

Designation

3

Statement of Interpreter / Signer / Communicator (if appropriate)

I have interpreted the information in sections 1, 2 and 5 to the patient/parent/guardian to the best of my ability and in a way in which I believe he/she can understand.

**Signed** (where possible)	Date

Name (PRINT)

	✔
Guardian/relative	☐
NHS Approved Translator	☐
Language Line	☐
Other: ______________	

4

Statement of Patient / Parent / Guardian

Please read this form carefully. If you wish to have a copy please ask. If you have any further questions, do ask - we are here to help you. You have the right to change your mind at any time, including after you have signed this form. Further information regarding the process of consent can be found in the NHS Grampian Consent Policy, which is available on NHS Grampian's public internet site or upon request from your clinician.

4a. I understand and agree to the procedure(s) described on this form.

4b. I understand that, although you cannot give me a guarantee that a particular person will perform the procedure, the person will have appropriate training and/or supervision.

4c. I understand that if my procedure(s) / treatment involves an anaesthetic, I will have the opportunity to discuss the details of this.

4d. I understand that an additional procedure(s) may be required and this has been explained in Section 1b.

4e. I understand that if unforeseen circumstances occur I may require alternative / additional procedure(s) / treatment but these will only be carried out to save my life or to prevent serious harm to my health.

**Patient's / Parent's / Guardian's signature**	Date

Name (PRINT)

Relationship to patient

	✔
• Young people/children may also like to sign here if they have given consent for their parent/ guardian to sign.	☐
• A witness should sign below if the patient is unable to sign but has indicated his or her consent.	☐
• Telephone consent can also be witnessed here.	☐

**Signed**	Witness name (PRINT)	Date

5

5a. Statement of health professional

In the course of the procedure(s)/treatment planned, blood transfusion is or may be required:

YES ☐ NO ☐

Approriate information regarding blood transfusion, such as that provided by the Scottish Blood Transfusion Service, has been given to the patient: YES ☐ NO ☐

Possible alternatives to transfusion have been discussed: YES ☐ NO ☐

Signed

Date

Name (PRINT)

Professional Reg. No

Designation

5b. Statement of Patient / Parent / Guardian

I understand and agree to blood transfusion as described: YES ☐ NO ☐

Patient's / Parent's / Guardian's signature

Date

Name (PRINT)

6

Confirmation of consent

(to be completed when the patient is admitted for the procedure/treatment, if the patient has signed the form in advance)

On behalf of the clinical team treating the patient, I confirm he/she has no further questions and wishes the procedure/treatment to go ahead.

Signed

Date

Name (PRINT)

Professional Reg. No

Designation

This leaflet is also available in larger print.

Other formats and languages can be supplied on request. Please call Quality Development on (01224) 554149 for a copy. Ask for leaflet 0409.

Feedback from the public helped us to develop this leaflet. If you have any comments on how we can improve it, please call (01224) 554149 to let us know.

Ward 203 Day Case Unit
Aberdeen Royal Infirmary
Leaflet supplied by:

revised July 2016
©NHS Grampian
Quality Development, Foresterhill

NHS Grampian

Simple eye treatment for blepharitis

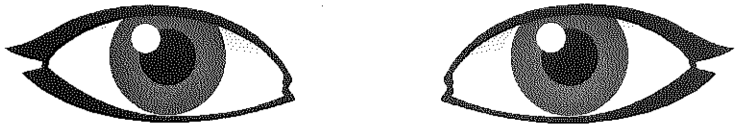

Information for patients and carers

OUTPATIENTS DEPARTMENT
DR GRAYS HOSPITAL

What is blepharitis?

Blepharitis is a common inflammatory eye disorder, which affects your eyelid margins and leaves debris on your eyelashes. If left untreated, the debris (germs living on the eyelids) can build up and look like dandruff, crusting at the roots of the lashes which can cause red sore eyes.

Why do I have blepharitis?

It just happens; it is not anything you have or have not done.

Will it cause problems?

Blepharitis is usually harmless, but can cause problems if left untreated. Some people have unpleasant irritation causing red eyes, and a "gritty" feeling due to the eyes being dry (lack of natural tears).

What is the treatment?

The treatment is described on the next page. The eyelid hygiene routine gradually helps remove the debris on your eyelashes. Over time, this will keep the condition under control, so be patient and keep up the good work.

It is important that you carry out this eyelid hygiene routine as instructed by your nurse to keep your blepharitis under control. Keeping it under control will also help ensure that planned eye surgery is not cancelled.

Your eyelid hygiene routine

To be done:

Once a day	☐
Twice a day	☐
Twice a week	☐

What do I need?

- fresh boiled water (allowed to cool)
- baby shampoo (any make)
- cotton buds
- mirror

Step 1

Mix **1 drop** of shampoo with **10 drops** of cool boiled water in a small container (such as an egg cup).

Step 2

Wash your hands before you start. Dip the cotton bud in the solution. With your eye closed, wipe the cotton bud across the base of your eyelashes from the nose side outwards using a firm action to help lift off the debris. (Use a mirror if doing this yourself or get someone to help you).

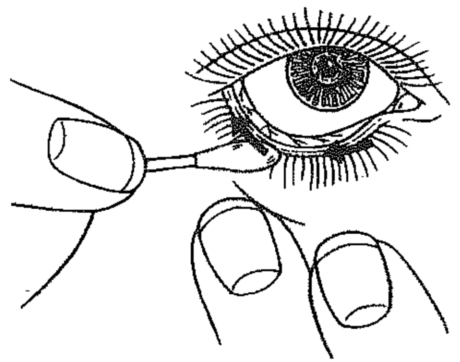

Diagram of eye wiping

Step 3

Repeat this action at least six times with a fresh cotton bud, cleaning both top and lower lashes.

Step 4

Now repeat this with your other eye starting at Step 1. If you have drops or ointment prescribed, put this in **after** your lid hygiene.

Contact advice numbers

Ward 203

Eye Inpatient Ward

☎ 0345 337 6360 ☎

This leaflet is also available in large print. Other formats and languages can be supplied on request. Please call Quality Development on (01224) 554149 for a copy. Ask for leaflet 0400.

Feedback from the public helped us to develop this leaflet. If you have any comments on how we can improve it, please call (01224) 554149 to let us know.

Ward 203
Aberdeen Royal Infirmary
Leaflet supplied by:

revised April 2014
©NHS Grampian
Quality Development, Foresterhill

thomsl 17/01/2018 14:56

NHS Grampian

Preparing for eye surgery

Information for patients and carers

Ophthalmic Unit, Ward 203 and Ward 301
Aberdeen Royal Infirmary

thomsl 17/01/2018 14:56

Ophthalmic Unit, Ward 203 and Ward 301

The following information is to help you prepare for your operation.

Please take time to read this booklet and note any questions you may have.

What is a cataract?

Cataract is a clouding of the lens of the eye. This causes hazy vision that may make it difficult for you to see well enough to carry out your normal activities. The purpose of the operation is to remove the cloudy lens (cataract) and replace it with a plastic lens (implant). This is a common operation but all operations have a small risk of complications developing. These will have been discussed with you by your doctor during your assessment visit.

Preparing for your operation

Please follow these instructions before coming into hospital.

- Take all your routine medication as normal. Please bring all your medications with you into hospital.
- Do not bring any valuables or money with you. NHS Grampian cannot accept any responsibility for items lost or stolen.
- Please have a bath/shower the night before or morning of your operation. Do not use any talc, deodorant, nail varnish, face make-up or face cream.
- Please contact the Ward 203 secretaries before your surgery day if you feel unwell (such as colds, flu, cold sores etc) as your operation may have to be rescheduled.

Ward 203, Secretaries
☎ (01224) 553217 or 552422 ☎

- If you are having morning surgery, please have a light breakfast (such as tea and toast) before you come into hospital.
- If your surgery is in the afternoon, you can have a normal breakfast and a morning snack before 11.00am.

- Please wear comfortable, loose fitting clothes; blouses / shirts with a front opening are best. Avoid wearing high neck jumpers. Please bring a dressing gown or cardigan with you.
- If you are having a general anaesthetic:
  - you will need an anaesthetic assessment before your operation.
  - please check during your assessment, when you should fast from (stop eating and drinking).
- Your visit to our department generally lasts around 6 hours. Please prepare for this length of stay.
- Due to limited space in our department, we ask that any relatives / friends do not wait in the ward during this appointment unless absolutely required to do so because of communication and / or condition difficulties you may have.
- Please ask your escort home to call the appropriate ward to arrange a time for you to be picked up.

Ward 203,
Inpatient Eye Ward
☎ (01224) 558977 ☎

Ward 301,
Short Stay Unit
☎ (01224) 551160 ☎

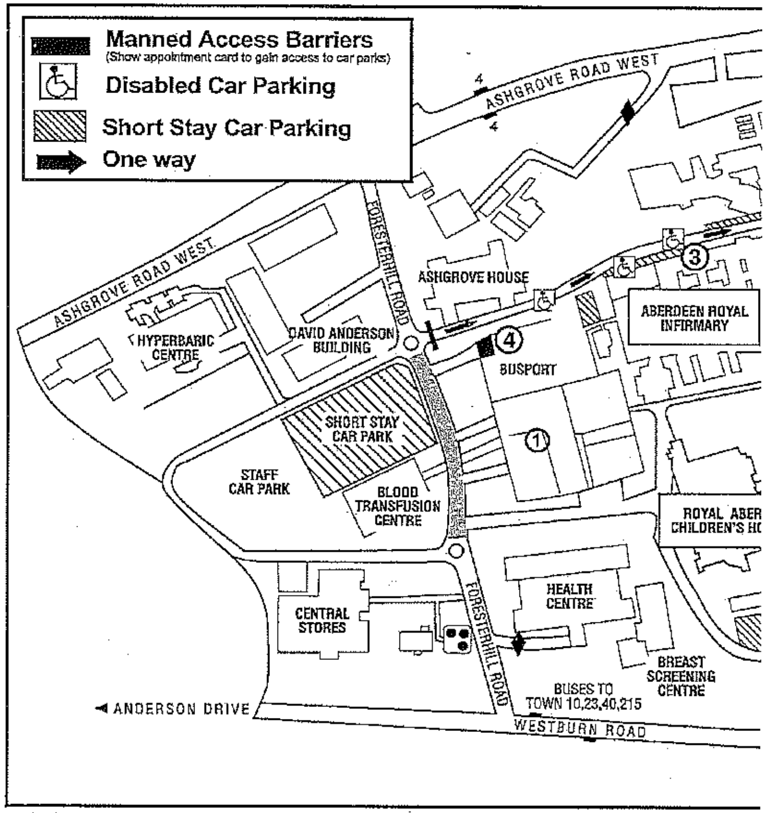

NOTE: Shaded section of Foresterhill Road: access for buses and emer
Short stay car park (for patients/visitors): 3 hours max stay. Please displ
Bus numbers 3, 4 (First) and 10, 59 (Stagecoach/Bluebird) use the busp

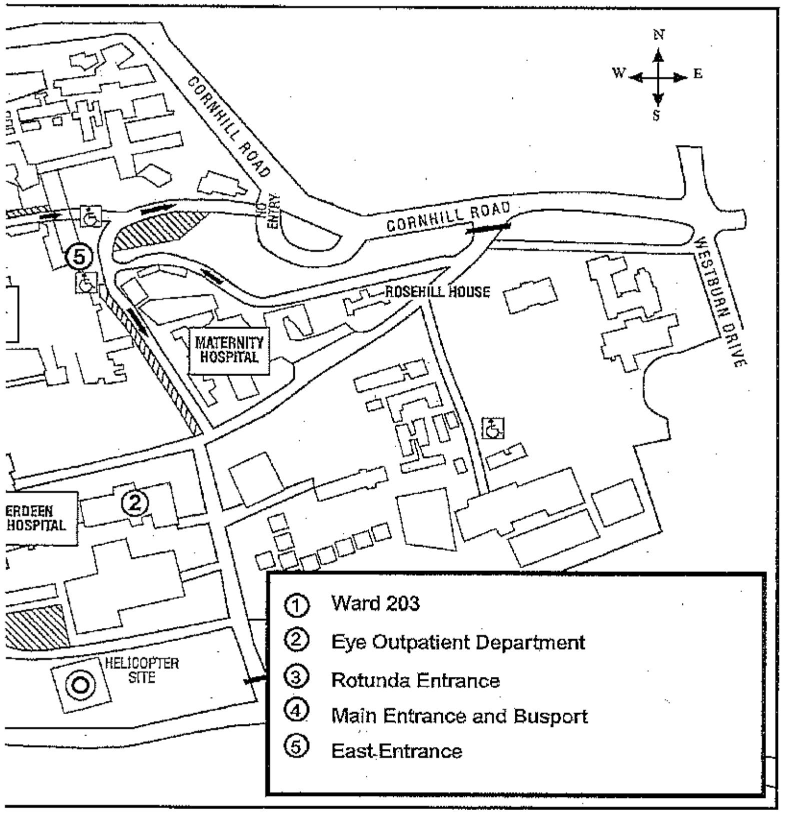

rgency vehicles only.
play ticket in your car.
port (④ on the map)

When you come into hospital

Please come directly to Ward 203 (on Level 2 of the Pink Zone) or Ward 301 (Level 1 of the Orange Zone) in Aberdeen Royal Infirmary. Please note that the wards are mixed sex areas. You may be nursed in a room with members of the opposite sex. Care will be taken to make sure that your privacy is maintained.

Admission to the ward

When you arrive in the ward, the receptionist will take your details and show you to your chair. The nurse looking after you will admit you for your operation, by asking you general questions and checking your pulse and blood pressure. You will be asked to change into a theatre gown.

Before your surgery your doctor may examine your eye and ask you some medical questions. The doctor will explain the operation to you and ask you to sign your consent form. Signing the form shows that you agree to and understand your surgery.

Your nurse will then bathe your eyes and put eyedrops in your eyes at regular intervals. This is done to enlarge the pupil in preparation for surgery.

You will be escorted to theatre by a nurse when it is time for your operation.

Your operation

The theatre nurse will welcome you and take over your care during your spell in theatre. A local anaesthetic is used to numb the eye, to prevent any discomfort during surgery. When your eye is numb, the nurse will take you into the operating room. You will be awake during the operation. You may be aware of bright lights and noises from the machines.

The nurse will stay with you and hold your hand during the procedure. If you should need to move or speak at all during the surgery, squeeze the nurse's hand, and he/she will ask the surgeon to stop.

During the surgery a light, pale green, cotton towel will be placed above your face and only the eye being operated on will be exposed. You will hear everything being said and sometimes the surgeon will explain to you what he/she is doing. You will not see what is happening.

The procedure will last approximately 20 to 30 minutes. Afterwards, a gauze pad and/or an eye shield will be placed over your eye. This dressing will normally remain on until that evening or the next morning.

A nurse from the ward will collect you and bring you back to the ward. The nurse will then check your pulse and blood pressure, and ask you to rest for about 30 minutes.

You can change back into your own clothes, and have something to eat and drink. Your nurse will prepare you for going home.

Going home

When it is time for you to go home, we will provide the following:

- a supply of eyedrops/ointment and an eye shield
- a letter for your GP
- information and advice on how you should look after your eye when you return home.
- your surgeon or a member of his/her team may examine your eye before you go home.

Note: you need to have your own surgical tape and cotton wool balls

thomsl 17/01/2018 14:56

Follow-up appointments

Your surgeon will decide when and by whom you need to be seen again after your surgery.

Most patients are asked to make an appointment with their local optician about 6 weeks after their operation. If this is the case for you, before you leave the ward we will give you a form for your optician to complete during this appointment. Please return the completed form to the Eye Outpatient Department in the pre-paid envelope provided.

Occasionally, your surgeon (or a member of his/her team) may ask to see you personally for your follow-up. If this is the case, we will organise this with you before you leave the ward.

This leaflet is also available in larger print.

Other formats can be supplied on request. Please call Quality Development on (01224) 554149 for a copy. Ask for leaflet 0093.

Feedback from the public helped us to develop this leaflet. If you have any comments on how we can improve it, please call (01224) 554149 to let us know.

Eye Inpatient Ward & Short Stay Unit
Aberdeen Royal Infirmary
Leaflet supplied by:

revised March 2014
©NHS Grampian
Quality Development, Foresterhill

NHS Grampian

Eye surgery aftercare

Information for patients and carers

Please bring this leaflet with you to hospital

Eye Inpatient Ward (Ward 203) and Short Stay Unit (Ward 301) Aberdeen Royal Infirmary

Although you have had a very short stay in hospital, the operation is still regarded as a major one. We would like to outline a few precautions for you to take.

Eye hygiene

If you find that your operated eye is sticky, it will need to be cleaned as follows:

1. Measure out a pint of boiled water. Leave to cool. (Any **unused** water can be stored in a clean air tight container in the fridge for 24 hours).
2. Wash your hands before cleaning your eye.
3. Using a small piece of cotton wool moistened with the above water, wipe your eyelids and lashes from the side of your eye nearest your nose to the outside. Do **not** touch the eye itself.
4. Use the cotton wool piece only once, then discard it. Repeat procedure until eye is clean.
5. If you are putting drops into your eye, do so **after** cleaning the eye.
6. If you are bathing both eyes, wash your hands between bathing each eye.

Eye drops

You will have been given eye drops before going home. It will have been fully explained to you and/or your relative **how** and **when** to put these in. If you finish your eye drops within 28 days, it is important to get a further supply from your GP.

Remember

- Wash your hands before using eye drops.
- If you have to use drops in both eyes, wash your hands **after** putting drops in **each** eye.
- Read the information on each bottle carefully, or have them marked so that you know which eye the drops are for and how often to use them, before putting in the drops.

Things to remember

- Wear your eye shield over your operated eye at night to protect your eye. Do this for 1 week.
- Wear your dark glasses, if you find that bright light makes your eye uncomfortable.
- Hand in your discharge letter to your GP's surgery as soon as possible.
- Avoid driving if you are using eye drops which dilate your pupil, until these drops are stopped. Your doctor or nurse will advise you of this.

Things to avoid

- Do not rub the operated eye.
- Avoid activities which have a risk of sustaining a direct blow to the eye (such as ball games, looking after small children, and swimming).
- Avoid getting substances into the eye (such as soap, shampoo, make up).

Pain relief

Your eye may ache when the numbing effect of the anaesthetic wears off. Pain relieving tablets of your choice can be taken. The eye should never be any more painful than it has been on the day or night of the operation.

Follow-up appointments

Your surgeon will decide when and by whom you need to be seen again after your surgery.

Most patients are asked to make an appointment with their local optician about 6 weeks after their operation. If this is the case for you, before you leave the ward we will give you a form for your optician to complete during this appointment.

Occasionally, your surgeon (or a member of his/her team) may ask to see you personally for your follow-up. If this is the case, we will organise this with you before you leave the ward.

Problems to watch out for

- Increasing eye pain.
- Increasing headache.
- Feeling sick.
- Increasingly sticky eye.
- Vision becoming much more blurred.

After your operation, if you have any problems with your eye, please call:

Eye Clinic
☎ 0345 337 6360 ☎

A nurse advisor is available
Monday to Friday, 9am to 5pm

If you have urgent difficulties outwith these hours please call:

Ward 203
☎ (01224) 552011 ☎

Please be aware that six weeks after your operation, your local optometrist (optician) can also provide free eye health care (paid for by the NHS) including emergency eye problems. They may also be able to help.

use the same bottle for the same eye.

- Your vision may become blurred for a short time after using your eye drops. If so, do not drive or operate machinery until your vision clears.
- Do not drive if you are using eye drops which dilate your pupils. You can start driving again when you have stopped using these drops. Your hospital doctor or nurse will have advised you of this.

Contact telephone number

Eye Clinic

☎ 0845 337 6360 ☎

A nurse adviser is available Monday to Friday 9am to 5pm

This leaflet is also available in larger print.

Other formats and languages can be supplied on request. Please call Quality Development on (01224) 554149 for a copy. Ask for leaflet 0445.

Feedback from the public helped us to develop this leaflet. If you have any comments on how we can improve it, please call (01224) 554149 to let us know.

Ward 203 Revised January 2014
Aberdeen Royal Infirmary
©NHS Grampian
Leaflet supplied by Quality Development, Foresterhill

NHS Grampian

How to use your eye drops

"Bridge of the nose method"

Aberdeen Royal Infirmary

If you need to use eye drops (for example, after surgery or admission to hospital), this leaflet will show you how to use them safely.

1. Holding the bottle between your thumb and your forefinger feel around the middle of the bottle for the squeezable area.

   Note: If putting drops into your right eye, hold bottle with left hand.

   If putting drops into your left eye, hold bottle with right hand.

2. Roll the bottle up your nose and rest the neck of the bottle on the area called the bridge of your nose, between your eyes (see diagram opposite).

3. With your spare hand, pull your lower eyelid down and look up towards the ceiling. Squeeze the bottle until one drop falls into your eye.

4. Once the drop has gone in, close your eye. Using a piece of cotton wool, wipe under your eyelid, with your eye closed, from the nose outwards, discarding the piece of cotton wool afterwards.

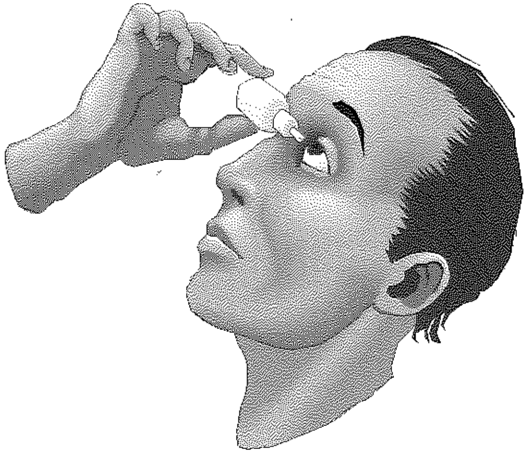

How to use your eye drops

Important reminders

- Remember to wash your hands before and after using the drops.
- Use the bottle of eye drops for 28 days only. If you still need to use eye drops after this time, please contact your GP for a repeat prescription.
- Use the eye drops as stated on the bottle or as your hospital doctor told you before you left hospital.
- Try to avoid letting the eye dropper touch your eye or anything else.
- Leave 5 minutes between using different drops.
- If you have been given one bottle for each eye, make sure you always
